# Temperature fluctuation and acute myocardial infarction in Beijing: an extended analysis of temperature ranges and differences

**DOI:** 10.3389/fpubh.2023.1287821

**Published:** 2023-12-11

**Authors:** Siqi Tang, Jia Fu, Yanbo Liu, Yakun Zhao, Yuxiong Chen, Yitao Han, Xinlong Zhao, Yijie Liu, Xiaofeng Jin, Zhongjie Fan

**Affiliations:** ^1^Department of Cardiology, Peking Union Medical College Hospital, Peking Union Medical College and Chinese Academy of Medical Sciences, Beijing, China; ^2^Department of Cardiology, Fuwai Yunnan Cardiovascular Hospital, Kunming, Yunnan, China; ^3^Department of International Medical Services, Peking Union Medical College Hospital, Peking Union Medical College and Chinese Academy of Medical Sciences, Beijing, China

**Keywords:** acute myocardial infarction, temperature range, temperature difference, age, sex

## Abstract

**Purpose:**

Few studies examined the relationship between temperature fluctuation metrics and acute myocardial infarction (AMI) hospitalizations within a single cohort. We aimed to expand knowledge on two basic measures: temperature range and difference.

**Methods:**

We conducted a time-series analysis on the correlations between temperature range (TR), daily mean temperature differences (DTDmean), and daily mean-maximum/minimum temperature differences (TDmax/min) and AMI hospitalizations, using data between 2013 and 2016 in Beijing, China. The effects of TR_n_ and DTDmean_n_ over n-day intervals were compared, respectively. Subgroup analysis by age and sex was performed.

**Results:**

A total of 81,029 AMI hospitalizations were included. TR_1_, TDmax, and TDmin were associated with AMI in J-shaped patterns. DTDmean_1_ was related to AMI in a U-shaped pattern. These correlations weakened for TR and DTDmean with longer exposure intervals. Extremely low (1st percentile) and high (5°C) DTDmean_1_ generated cumulative relative risk (CRR) of 2.73 (95% CI: 1.56–4.79) and 2.15 (95% CI: 1.54–3.01). Extremely high TR_1_, TDmax, and TDmin (99th percentile) correlated with CRR of 2.00 (95% CI: 1.73–2.85), 1.71 (95% CI: 1.40–2.09), and 2.73 (95% CI: 2.04–3.66), respectively. Those aged 20–64 had higher risks with large TR_1_, TDmax, and TDmin, while older individuals were more affected by negative DTDmean_1_. DTDmean_1_ was associated with a higher AMI risk in females.

**Conclusion:**

Temperature fluctuations were linked to increased AMI hospitalizations, with low-temperature extremes having a more pronounced effect. Females and the older adult were more susceptible to daily mean temperature variations, while younger individuals were more affected by larger temperature ranges.

## 1 Introduction

Acute myocardial infarction (AMI) continues to carry a substantial health burden worldwide (1). Ambient temperature has been established to mediate an elevated risk of AMI (2–4). However, long-term cold and heat exposure generates biological and behavioral acclimatization, thus modifying the influence of absolute temperature (5, 6). For example, early studies showed a more pronounced effect of lower temperatures during warmer years (4). Prior studies linked short-term cold exposure to increased inflammation and hyper-coagulation, predisposing individuals to AMI (7). Also, temperature fluctuations disrupt autonomic function by elevating blood pressure and heart rate (8), exaggerating the myocardial oxygen demand–supply imbalance in those with preexisting coronary lesions (9).

Recent data highlighted the adverse effect of temperature fluctuation using an array of metrics (10–16). Intra-day temperature range and day-to-day temperature difference are commonly studied metrics, capturing different aspects of temperature fluctuations. In prior studies, greater neighboring day temperature differences were linked to increased cardiovascular visits and hospitalizations (17). A larger diurnal temperature range contributed to increased coronary heart disease-related death (18) and out-of-hospital cardiac arrests (19). However, few studies examined the impacts of temperature range differences within a single cohort. Also, it remains unclear whether 1-day intervals are the optimal observation periods for assessing day-to-day temperature differences and temperature ranges.

In this time-series study, we used registry data for all AMI hospitalization in Beijing, China, a heavily populated city with a humid continental climate. We performed an extended analysis on two basic measures: temperature range and daily mean temperature difference, aiming to understand their effects on AMI hospitalization and explored the optimal observation intervals. Additionally, we sought to identify susceptible age and sex subgroups for different patterns of temperature fluctuation, thus informing targeted prevention strategies.

## 2 Materials and methods

### 2.1 Data collection

We collected all cases of hospital admission for AMI between January 1st, 2013 and December 31st, 2016 in Beijing. Data were obtained from the Beijing Municipal Health Commission Information Center. Anonymous demographic and residential information was collected, including the institute of admission, date of onset, gender, age, primary diagnosis, and comorbidities. Birthplace, current residential address, and workplace address were used to exclusively include patients who resided in Beijing. Patients aged between 20–74 years old were included in the analysis. AMI hospitalization was identified by the primary diagnostic code of I21–I22, according to the International Classification of Diseases 10th revision (ICD-10). The study was approved by the Peking Union Medical College Hospital (PUMCH) Institutional Review Board.

City-level meteorological data, including daily mean temperature (Tmean), maximum temperature (Tmax), minimum temperature (Tmin), air pressure, relative humidity (RH), and wind speed (WS) were recorded by the China Meteorological Administration (CMA). The data were collected by a stationary monitoring station located near the city center (Station code: 54511). Air pollutant data were included as confounding factors, which were collected from 35 monitoring stations across Beijing. This included the hourly concentration of both the particulate (PM_2.5_, PM_10_) and gaseous air pollutants (SO_2_, NO_2_, O_3_, CO). To address daily variations in a set of pollutants, we calculated the Air Quality Index (AQI) which combines the effects of six common air pollutants for the same period. Influenza was independently associated with an increased risk of AMI in the previous study (20). We collected data on influenza epidemic (IF), which was defined as when the positive rate of influenza isolation in any given week exceeded 20% of the maximum weekly positive rate of influenza isolation in the whole surveillance season (from the 27th week of the previous year to the 26th week of the following year) in northern China (13, 14). The influenza surveillance data were obtained from the Chinese National Influenza Center.^1^

### 2.2 Temperature variables

We estimated the influence of temperature range (TR_n_) and daily mean temperature difference (DTDmean_n_) on the count of AMI hospital admissions. TR_n_ represents the difference between maximal temperature and minimum temperature over an n-day period, ie. Tmax minus Tmin over n days. DTDmean_n_ was derived from the mean temperature difference between the current day and n day prior. To further understand the intra-day cold and heat effects, we investigated intra-day cold and heat effects using daily maximum/minimum and mean temperature difference (TDmax/TDmin) which represented the same-day Tmax/ Tmin minus mean temperature.

### 2.3 Statistical analysis

Distributed Lag Non-linear Model was established to fit the nonlinear effect and lag effect of independent variables (12–14). Long-term and seasonal trends were controlled using a natural cubic spline with 7 degrees of freedom (df) for the time. We defined the seasons based on astronomical seasons, which were spring (March 20th to March 21st), summer (June 20th to June 21st), autumn (September 22nd to September 23rd), and winter (December 20th to December 21st). Wind speed, air pressure, relative humidity, and Air Quality Index were adjusted using a natural cubic spline with 3 df. Public holiday (PH) and day of the week (DOW) were adjusted for their impacts on the behavioral patterns. The full model was as below:

$$\begin{array}{c} \log\left[E\left(Y_{t}\right)\right]=\alpha +cb\left(Temp_{t},lag,df=4\right)+ns\left(Time,df=7\;per\;year\right)+ns\left(WS,df=3\right)+\\ ns\left(AP,df=3\right)+ns\left(RH,df=3\right)+ns\left(AQI,df=3\right)+\beta_{1}*DOW+\beta_{2}*IF+\beta_{3}*PH \end{array}$$

*E (Yt)* denotes the number of AMI hospital admissions on day *t*. *α* and *β* were the model intercept and regression coefficient, respectively. *cb* represents the cross-basis function and ns indicates the natural cubic spline function. *Tmp* refers to different temperature variables. *Time* refers to the time to control the season and long-term trends. *df* represents the degree of freedom.

3D maps depicted the overall relationship between temperature variables and AMI relative risks (RR) over 21 lag days (lag). We plotted the lag-response curves of temperature variables at 1^st^, 5^th^, 95^th^, and 99^th^, respectively. Twenty-one-day cumulative relative risk (CRR), the sum of the relative risk of each lag day within 21 days, was calculated to assess the overall effects of temperature variables and control the possible harvesting effect. The most moderate temperature difference (MMTD) was defined as the optimal temperature difference that carries the lowest risk of AMI, which served as the reference when evaluating relative risks. We performed stratification analysis by gender and age (<65 vs. ≥65 years old) and tested the reliability of the results. Statistical analyses were conducted in R software (R x64 v3.4.2) using “mgcv” and “dlnm” packages. A two-sided *p* value of 0.05 was considered statistically significant.

## 3 Results

### 3.1 Descriptive analysis

Between 2013 and 2016, we identified a total of 81,029 hospitalizations for AMI, with 55,669 (68.7%) being male and 36,989 (45.6%) under the age of 65. We observed a trend of increase in hospital admissions for AMI. Supplementary Table S1 shows the descriptive statistics of the study population and meteorological data in Beijing, China.

The maximum, mean, and minimum daily temperatures were 19.01°C, 12.94°C, and 7.19°C, respectively. The mean Air Quality Index, relative humidity, wind speed, and air pressure were 123.65 ± 75.17, 53.43 ± 19.86, 9.29 ± 4.75 m/s, 53.43 ± 19.86%, and 1016.555 ± 10.17 hPa, respectively. Summary statistics for meteorological and air pollution are summarized in Supplementary Table S2.

### 3.2 Cumulated relative risk

**Figure 1:**
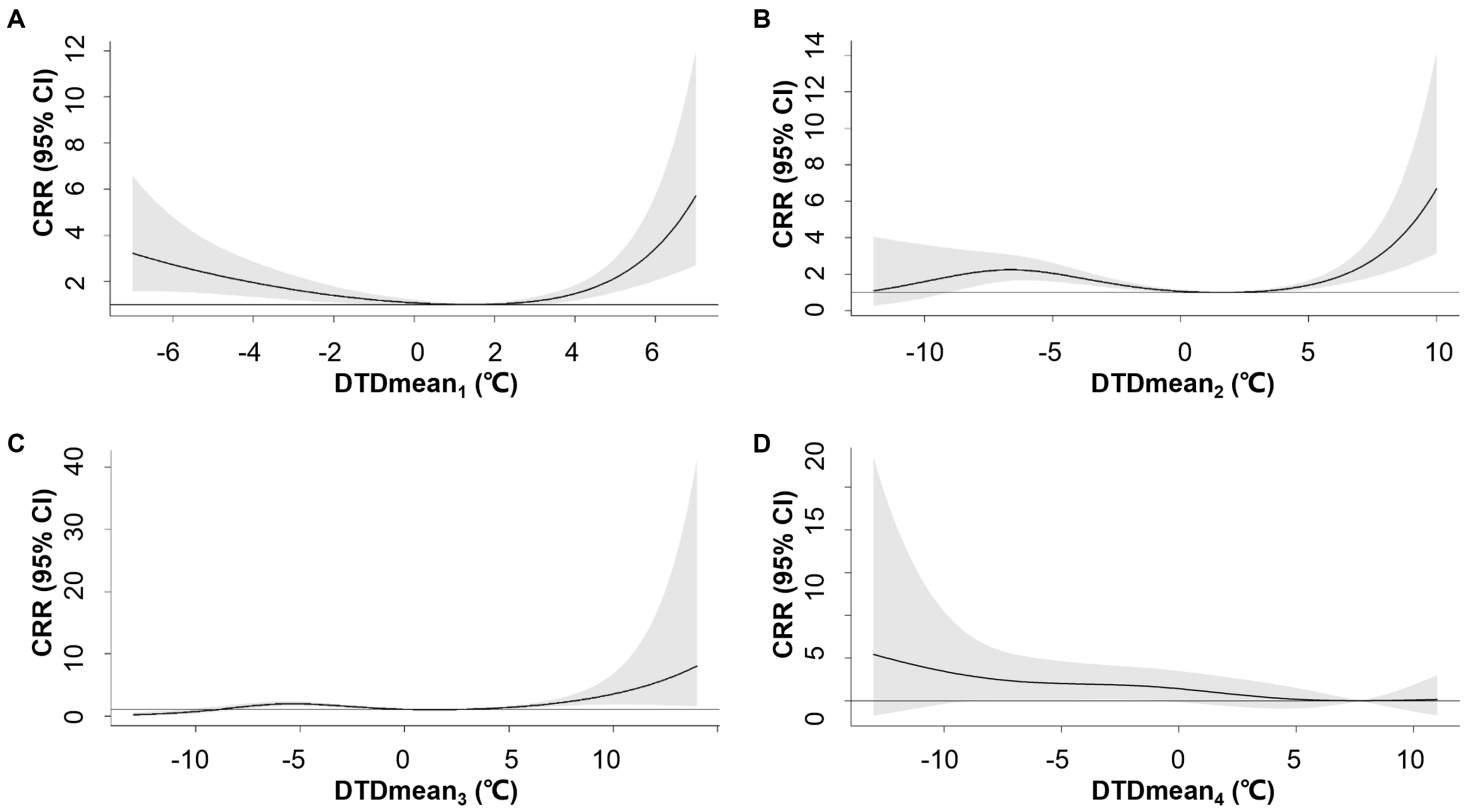
Overall exposure-response associations between neighboring-day mean temperature difference (DTDmean) over 1–4 days **(A–D)** and cumulative relative risks (CRR) for AMI hospitalization. Shaded areas represent 95% CI.

Compared to DTDmean_1_, longer exposure intervals (DTDmean_2-4_) attenuated the association between DTDmean and AMI hospitalization (Figure 1). Notably, DTDmean_4_ did not increase the risk of AMI hospitalization. The exposure-response association between DTDmean_1_ and AMI hospitalization was U-shaped. The AMI hospitalization risk reached a nadir at 1.4°C, namely MMTD for DTDmean_1_, and marked increases in risk were observed at both low and high DTDmean_1_. On days with DTDmean_1_ values at the 1st percentile (−6°C) and 99^th^ percentile (5°C), the CRR reached 2.73 (95% confidence interval, CI: 1.56–4.79) and 2.15 (95% CI: 1.54–3.01), respectively (Supplementary Table S3).

**Figure 2:**
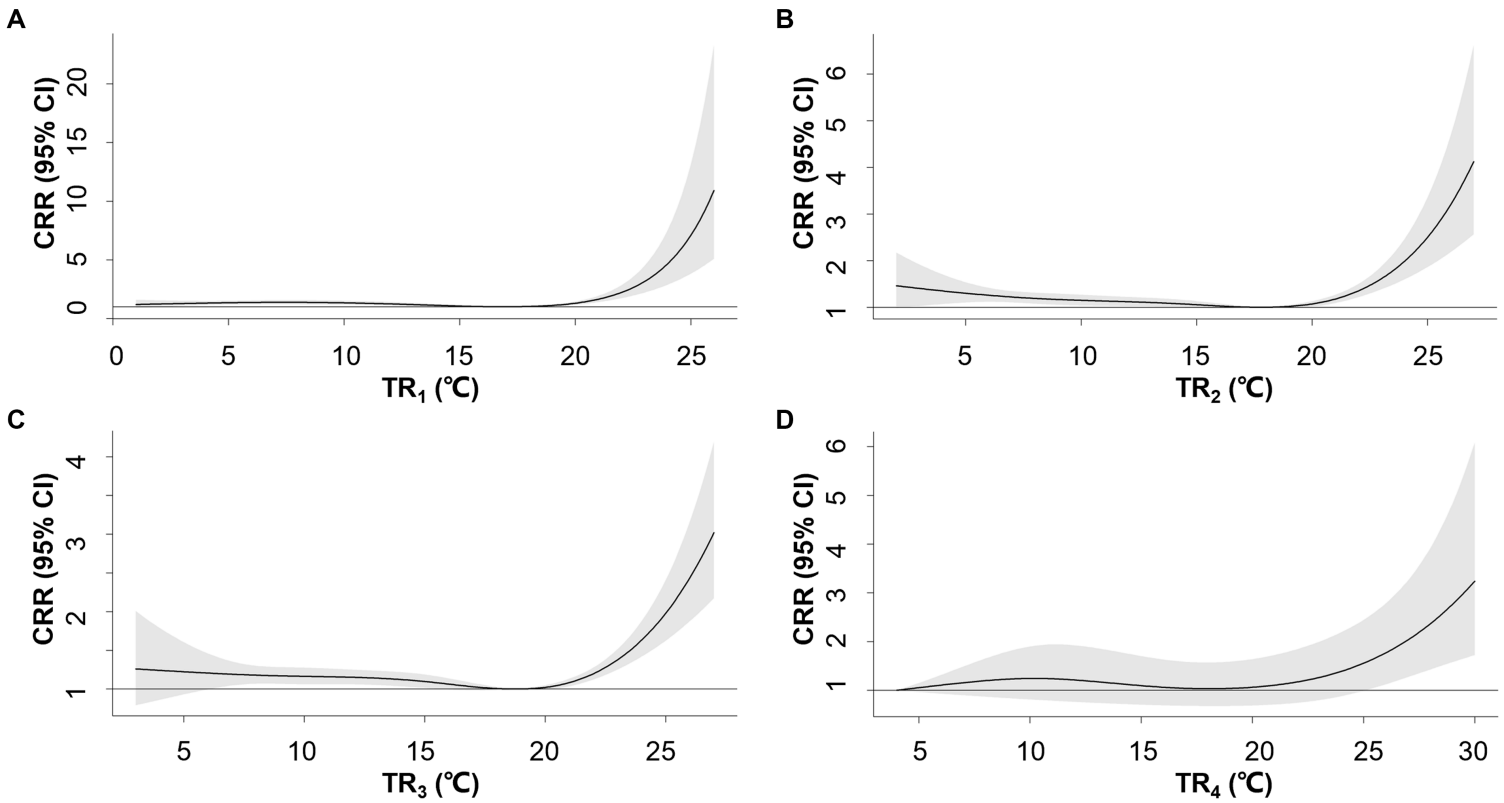
Overall exposure-response associations between temperature range (TR) over 1–4 days **(A–D)** and cumulative relative risks (CRR) for AMI hospitalization. Shaded areas represent 95% CI.

The association between TR and AMI hospitalization weakened with longer exposure intervals when comparing TR_1-5_ (Figure 2). No significant relationship was observed between TR_5_ and the risk of AMI hospitalization. The association between TR_1_ and the risk of AMI hospitalization exhibited a J-shaped pattern, where the risk increased when TR_1_ exceeded 16.9°C. TR_1_ at the 95th (19°C) and 99th percentile (22°C) were associated with CRRs of 1.12 (95% CI: 1.04–1.20) and 2.0 (95% CI: 1.73–2.85), respectively (Supplementary Table S4).

**Figure 3:**
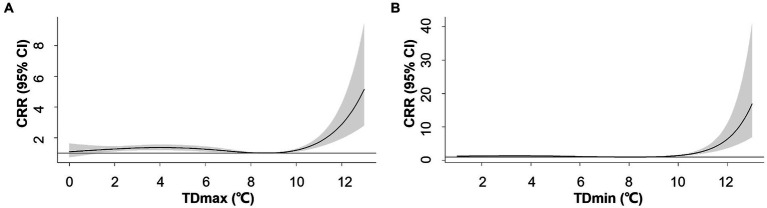
Overall exposure-response associations between maximum-mean temperature difference (TDmax, **A**), mean-minimum temperature difference (TDmin, **B**) and cumulative relative risks (CRR) for AMI hospitalization. Shaded areas represent 95% CI.

The overall patterns of TDmax/TDmin-AMI hospitalization association were similar to TR_1_ with varying magnitude of association (Figure 3). The positive associations were observed when TDmin and TDmax exceeded 8.1°C and 8.6°C, respectively. For TDmax at the 95^th^ percentile (10°C) and 99^th^ percentile (11°C), the CRRs were 1.18 (95% CI: 1.09–1.29) and 1.71 (95% CI: 1.40–2.09), respectively (Supplementary Table S5). For TDmin at the 95th percentile (9°C) and 99^th^ percentile (11°C), the corresponding CRRs were 1.07 (95% CI: 1.00–1.15) and 2.73 (95% CI: 2.04–3.66), respectively (Supplementary Table S6).

### 3.3 Single-day Lag effects

Negative (temperature decline) and positive (temperature rise) DTDmean_1_ showed different patterns of lag effects (Supplementary Figure S1). The RR of DTDmean_1_ at −6°C (1st percentile) and − 4°C (5th percentile) peaked on lag day 10 and yielded a lag effect throughout lag day 3 to 18 and lag day 3 to 21, respectively. For positive DTDmean_1_, we observed delayed peak lag effects. The effect of DTDmean_1_ at 5°C (99^th^ percentile) and 4°C (95^th^ percentile) both extended from lag day 1 to 21, with the RR peaked on lag day 21.

Similar lag patterns were observed among TR_1_, TDmax, and TDmin at the 99^th^ percentile, which lasted for 20 days and peaked on lag day 2–3 (Supplementary Figure S2). No significant associations were found for TR_1_ and TDmax at the 95th percentile. For TDmin at the 95th percentile (9°C), the lag effect was only significant on lag day 0 and 1, reaching the peak RR on lag day 0.

Supplementary Figure S3 shows the 3D mapping of the association between relative risk AMI hospitalization and DTDmean_1_, TR_1_, TDmax, and TDmin over the 21-day lag period.

### 3.4 Age- and sex-specific effect

Compared to their younger counterparts, individuals aged over 65 years were at higher risk of AMI hospitalization on days with negative DTDmean_1_ (−6°C) [3.04 (95% CI: 1.48–6.22) vs. 2.40 (95% CI: 1.17–4.92)]. Negative DTD mean_1_ was more strongly associated with the AMI hospitalization risk in females than males [3.40 (95% CI: 1.42–8.12) vs. 2.46 (95% CI: 1.29–4.67)] (Supplementary Figure S4). Comparable CRRs were observed among age and sex subgroups with positive DTDmean_1_ (Supplementary Table S3).

**Figure 4:**
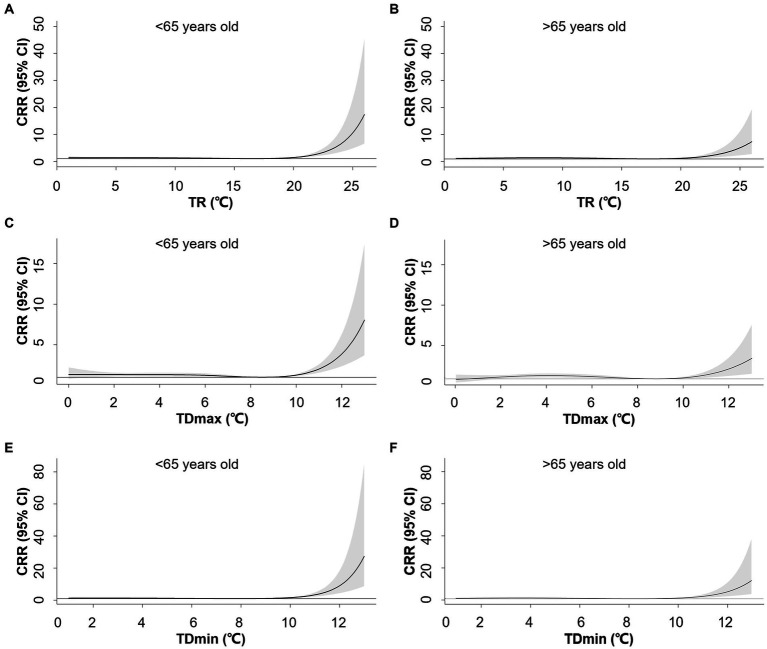
Exposure-response associations between 1-day temperature range (TR_1_, **A,B**), maximum-mean temperature difference (TDmax, **C,D**), mean-minimum temperature difference (TDmin, **E,F**) and cumulative relative risks (CRR) for AMI hospitalization stratified by age. Shaded areas represent 95% CI.

In contrast, the associations between TR_1_, TDmax, and TDmin were attenuated at older ages. For TR_1_ at the 99^th^ (22°C) percentile, the CRR was 2.69 (95% CI: 1.07–3.68) for the younger group and 1.90 (95% CI: 1.38–2.60) for the older group. TDmax at the 99^th^ percentile (11°C) corresponded to a CRR of 2.01 (95% CI: 1.56–2.59) for the younger group and 1.49 (95% CI: 1.15–1.92) for the older group. Specifically, TDmin yielded a CRR of 3.36 (95% CI: 2.32–4.87) for the younger group and 2.29 (95% CI: 1.58–3.33) for the older group at the 99^th^ percentile (11°C) (Figure 4). Slightly increased risks were noted for females with greater TR_1_, TDmax, and TDmin (Supplementary Figure S5 and Supplementary Tables S4–S6).

## 4 Discussion

In this study, we conducted a comprehensive analysis of the impacts of temperature range and differences on AMI hospitalization in Beijing, China. Increased temperature range and day-to-day temperature difference were both associated with higher AMI risk with the optimal 1-day observation interval. Specifically, the older adult population was more susceptible to day-to-day temperature differences, while the younger population was subject to larger temperature ranges, represented by TR1, TDmin, and TDmax. Females were more affected by neighboring-day temperature declines.

Previous studies have linked neighboring-day temperature differences to coronary heart disease. In a study conducted in Brisbane and Los Angeles, a temperature drop and increase of more than 3°C between neighboring days were associated with a relative risk of 1.252 (95%CI: 1.131–1.386) and 1.35 (95% CI: 1.033, 1.772) for cardiovascular deaths during summer (21). Similarly, Shi et al. demonstrated a V-shaped relationship between neighboring day temperature differences and cardiovascular visits and hospitalizations in northwest China (17). These findings suggest that day-to-day temperature change, regardless of the direction of the change, contributes to increased cardiovascular risks.

The diurnal temperature range (DTR), in our setting, TR_1_, was also related to increased AMI risks. A study in Shanghai, China showed that a 1°C increment in the DTR yielded a 2.46% increase in coronary heart disease-related death (18). In a New York-state-based study, DTR was positively associated with AMI risk (10). In a Japanese study between 2005 and 2013, DTR was related to increased risks of out-of-hospital cardiac arrests, but less significant when compared to mean temperature (19). However, Lim et al. found an adverse effect between DTR and cardiovascular admissions, but no effect on AMI in Korea between 2003 and 2006 (22). In addition, we examined the influence of cold and heat effects by incorporating TDmin and TDmax. We found that TDmin had a more substantial impact on AMI risks, indicating a stronger association with cold temperature extremes.

Few studies examined the impacts of observation intervals, primarily focusing on 1 to 2 days of variability. A Brazilian study between 2000 and 2015 observed the most significant effect of temperature variability (measured as the standard deviation of daily minimum and maximum temperatures) on ischemic heart disease risk during 0–1 day, with the effect diminishing over 0–4 days (23). Despite the strongest estimates for 1-day exposure, we also identified a minor yet statistically significant effect of temperature fluctuation over 2 to 3 days. These results are consistent with the findings of Pearce et al., who showed that temperature trajectories in preceding days modified the associations between daily temperature and mortality in Melbourne, Australia (24).

The effect of temperature fluctuations on the risk of AMI hospitalization varied by age. In line with the previous studies, the older adult population, characterized by diminished thermoregulatory capacity, displayed greater susceptibility to day-to-day temperature variations (10, 22). Contrary to prior findings, we observed that younger participants were more prone to significant intra-day temperature ranges. In addition, low-temperature extremes, represented by TDmin may contribute to a more pronounced influence on AMI. This discrepancy might be attributed to the lower age cut-off compared to the previous studies (75 years) (22, 25), which consisted mainly of the working population. We hypothesized that daily commutes led to greater temperature fluctuation exposure, resulting in a higher risk of AMI in the younger population. Additional behavioral studies are needed to investigate these discrepancies.

Existing data demonstrated inconsistent modification effects by sex. In the WHO MONICA project between 1980 and 1995, females living in warm climates exhibited higher coronary event rates during cold periods (2). However, no gender differences in the seasonality of AMI hospitalization in the Taiwan study between 1997 and 2011 (26). An hour-to-hour study in Queensland, Australia, showed that elevated risks occurred more acutely in males following extreme cold exposure (9 h in males vs. 19 h in females) (27). Physiological studies suggest that females had weaker sweating responses, greater heat loss due to a larger surface area, and periodic thermoregulation due to menstrual cycles (28, 29). However, geographic differences may account for the divergence across the study, involving biological and habitual adaptations. For example, in the Brazilian study, males engaged in more outdoor activities, possibly making them more susceptible to ambient temperature (23).

Our study focused on observations in Beijing, a densely populated temperate city. We investigated the effects of DTDmean and TR over 1–5 days’ exposure and their differential effects on age and sex. However, our study has several limitations. Firstly, as a single-city study, the findings may not apply to regions with different climate types. Secondly, the data might not represent individual-level exposure, as indoor temperature exposure was not analyzed. Lastly, using data from city-wide monitoring stations introduces potential measurement errors that cannot be fully eliminated.

## 5 Conclusion

Temperature fluctuations were linked to increased AMI hospitalizations, with low-temperature extremes having a more pronounced effect. Females and the older adult were more susceptible to daily mean temperature variations, while younger individuals were more affected by larger temperature ranges.

## Data availability statement

The original contributions presented in the study are included in the article/Supplementary material, further inquiries can be directed to the corresponding authors.

## Ethics statement

The studies involving humans were approved by Peking Union Medical College Hospital (PUMCH) Institutional Review Board. The studies were conducted in accordance with the local legislation and institutional requirements. Written informed consent for participation was not required from the participants or the participants' legal guardians/next of kin in accordance with the national legislation and institutional requirements.

## Author contributions

ST: Formal analysis, Writing – original draft, Writing – review & editing. JF: Conceptualization, Data curation, Formal analysis, Writing – original draft. YL: Formal analysis, Writing – review & editing. YZ: Data curation, Writing – review & editing. YC: Methodology, Writing – original draft. YH: Software, Writing – original draft. XZ: Project administration, Writing – review & editing. YL: Software, Writing – original draft. XJ: Investigation, Project administration, Supervision, Validation, Visualization, Writing – review & editing. ZF: Funding acquisition, Project administration, Supervision, Validation, Writing – review & editing.

## Footnotes

^1^ https://ivdc.chinacdc.cn/cnic/zyzx/lgzb
