# Supplementary material for: Temperature fluctuation and acute myocardial infarction in Beijing: an extended analysis of temperature ranges and differences
Source: Front Public Health. 2023 Dec 11;11:1287821. doi: 10.3389/fpubh.2023.1287821 (PMC10749349; doi:10.3389/fpubh.2023.1287821)
Supplement: Supplementary file 1 [file Data_Sheet_1.pdf]

*Supplementary Material*

# 1 Supplementary Tables

**Supplementary Table 1. Descriptive statistics of study population: AMI hospitalization in Beijing, China, 2013 to 2016**

| | | Daily AMI hospitalization counts | | |
|---|---|---|---|---|
| | **No. of cases** | **Min*** | **Median** | **Max**** |
| Total | 81,029 | 23 | 54 | 152 |
| Age | | | | |
| <65 | 36,989 | 7 | 25 | 70 |
| ≥65 | 44,040 | 9 | 29 | 93 |
| Gender | | | | |
| Male | 55,669 | 13 | 37 | 104 |
| Female | 25,360 | 3 | 17 | 57 |
| Year | | | | |
| 2013 | 18,367 | 23 | 49 | 90 |
| 2014 | 18,998 | 25 | 52 | 83 |
| 2015 | 20,442 | 24 | 56 | 94 |
| 2016 | 23,222 | 31 | 61 | 152 |

* minimum, ** maximum

**Supplementary Table 2. Summary Statistics for Meteorology and Air Pollutants (Data on 1461 consecutive days**

| Parameters | Mean (SD) | Min* | P25** | P50** | P75** | Max*** |
|---|---|---|---|---|---|---|
| Temperature measures | | | | | | |
| Neighboring-day mean temperature difference ($DTDmean_1$), ℃ | 0 ± 2.26 | 7 | 1 | 0 | 1 | 7 |
| 2-day mean temperature difference ($DTDmean_2$), ℃ | 0 ± 2.94 | 12 | 2 | 0 | 2 | 10 |
| 3-day mean temperature difference ($DTDmean_3$), ℃ | 0 ± 3.22 | 13 | 2 | 0 | 2 | 14 |
| Mean temperature (Tmean), ℃ | 12.88 ± 11.17 | 16 | 2 | 14 | 23 | 32 |
| Maximum temperature (Tmax), ℃ | 18.95 ± 11.39 | 13 | 8 | 21 | 29 | 42 |
| Minimum temperature (Tmin), ℃ | 7.13 ± 11.34 | 17 | 3 | 8 | 18 | 27 |
| 1-day temperature range ($TR_1$), ℃ | 11.82 ± 4.31 | 1 | 9 | 12 | 15 | 26 |
| 2-day temperature range ($TR_2$), ℃ | 14.27 ± 3.95 | 2 | 12 | 14 | 17 | 27 |
| 3-day temperature range ($TR_3$), ℃ | 15.76 ± 3.73 | 3 | 13 | 16 | 18 | 27 |
| Maximum-mean temperature difference (TDmax), ℃ | 6.07 ± 2.23 | 0 | 5 | 6 | 8 | 13 |
| Mean-minimum temperature difference (TDmin), ℃ | 5.75 ± 2.15 | 1 | 4 | 6 | 7 | 13 |
| Other meteorological variables | | | | | | |
| Air Quality Index | 123.65 ± 75.17 | 23 | 68 | 104 | 159 | 485 |
| Relative humidity, % | 53.43 ± 19.86 | 8 | 38 | 53 | 69 | 97 |
| Wind speed, m/s | 9.29 ± 4.75 | 3 | 6 | 8 | 11 | 34 |
| Air pressure, hPa | 1016.56 ± 10.17 | 994 | 1008 | 1016 | 1025 | 1044 |

* minimum, ** the 25th, 50th (median) and 75th percentile, respectively, *** maximum

**Supplementary Table 3. Exposure-response associations between $DTDmean_1$ and CRR (95% CI) over 21 days for AMI hospitalization stratified by age and sex.**

| | CRR (95% CI) | | | | |
|---|---|---|---|---|---|
| **Percentiles** | Overall | <65 years old | ≥65 years old | Male | Female |
| **P1(-6°C)** | 2.73 (1.56, 4.79) | 2.40 (1.17, 4.92) | 3.04 (1.48, 6.22) | 2.46 (1.29, 4.67) | 3.04 (1.42, 8.12) |
| **P5(-4°C)** | 1.95 (1.33, 2.86) | 1.69 (1.04, 2.75) | 2.20 (1.35, 3.58) | 1.82 (1.18, 2.81) | 2.27 (1.25, 4.10) |
| **P10(-3°C)** | 1.65 (1.20, 2.27) | 1.45 (0.96, 2.18) | 1.83 (1.22, 2.75) | 1.57 (1.09, 2.25) | 1.83 (1.12, 3.01) |
| **P25(-1°C)** | 1.20 (1.00, 1.44) | 1.15 (0.91, 1.44) | 1.25 (0.99, 1.58) | 1.19 (0.97, 1.46) | 1.24 (0.93, 1.64) |
| **P75(1°C)** | 1.01 (0.97, 1.05) | 1.00 (0.96, 1.06) | 1.01 (0.96, 1.06) | 1.01 (0.96, 1.05) | 1.00 (0.94, 1.07) |
| **P90(3°C)** | 1.14 (0.99, 1.33) | 1.13 (0.93, 1.36) | 1.16 (0.96, 1.40) | 1.13 (0.96, 1.34) | 1.17 (0.93, 1.48) |
| **P95(4°C)** | 1.47 (1.18, 1.84) | 1.42 (1.07, 1.89) | 1.51 (1.14, 2.00) | 1.44 (1.12, 1.86) | 1.52 (1.09, 2.17) |
| **P99(5°C)** | 2.15 (1.54, 3.01) | 2.03 (1.32, 3.12) | 2.23 (1.46, 3.42) | 2.09 (1.43, 3.07) | 2.28 (1.35, 3.83) |

CRR, cumulative relative risks; CI, confidence interval

**Supplementary Table 4. Exposure-response associations between $TR_1$ and CRR (95% CI) over 21 days for AMI hospitalization stratified by age and sex.**

| | CRR (95% CI) | | | | |
|---|---|---|---|---|---|
| **Percentiles** | Overall | <65 years old | ≥65 years old | Male | Female |
| **P75(15°C)** | 1.06 (0.99, 1.14) | 1.04 (0.95, 1.13) | 1.08 (0.99, 1.18) | 1.03 (0.96, 1.12) | 1.12 (1.01, 1.25) |
| **P90(17°C)** | 1.00 (1.00, 1.00) | 1.00 (1.00, 1.01) | 1.00 (0.99, 1.00) | 1.00 (1.00, 1.01) | 1.00 (0.99. 1.00) |
| **P95(19°C)** | 1.12 (1.04, 1.20) | 1.17 (1.08, 1.28) | 1.07 (0.98, 1.17) | 1.14 (1.05, 1.23) | 1.08 (0.97, 1.19) |
| **P99(22°C)** | 2.00 (1.73, 2.85) | 2.69 (1.97, 3.68) | 1.90 (1.38, 2.60) | 2.20 (1.66, 2.91) | 2.27 (1.55, 3.32) |

CRR, cumulative relative risks; CI, confidence interval

**Supplementary Table 5. Exposure-response associations between TDmax and CRR (95% CI) over 21 days for AMI hospitalization stratified by age and sex.**

| | CRR (95% CI) | | | | |
|---|---|---|---|---|---|
| **Percentiles** | Overall | <65 years old | ≥65 years old | Male | Female |
| **P75(8°C)** | 1.03 (0.98, 1.07) | 1.02 (0.97, 1.07) | 1.03 (0.98, 1.09) | 1.01 (0.96, 1.06) | 1.06 (1.00, 1.13) |
| **P90(9°C)** | 1.01 (0.99, 1.04) | 1.02 (0.99, 1.05) | 1.00 (0.97, 1.03) | 1.02 (0.99, 1.05) | 0.99 (0.95. 1.03) |
| **P95(10°C)** | 1.18 (1.09, 1.29) | 1.26 (1.13, 1.40) | 1.12 (1.01, 1.25) | 1.21 (1.10, 1.33) | 1.14 (1.00, 1.29) |
| **P99(11°C)** | 1.71 (1.40, 2.09) | 2.01 (1.56, 2.59) | 1.49 (1.15, 1.92) | 1.71 (1.36, 2.15) | 1.70 (1.25, 2.32) |

CRR, cumulative relative risks; CI, confidence interval

**Supplementary Table 6. Exposure-response associations between TDmin and CRR (95% CI) over 21 days for AMI hospitalization stratified by age and sex.**

| | CRR (95% CI) | | | | |
|---|---|---|---|---|---|
| **Percentiles** | Overall | <65 years old | ≥65 years old | Male | Female |
| **P75(7°C)** | 1.06 (0.99, 1.14) | 1.03 (0.95, 1.13) | 1.09 (1.00, 1.19) | 1.04 (0.96, 1.13) | 1.11 (0.99, 1.23) |
| **P90(9°C)** | 1.07 (1.00, 1.15) | 1.11 (1.02, 1.21) | 1.04 (0.96, 1.14) | 1.09 (1.01, 1.18) | 1.04 (0.94, 1.16) |
| **P95(9°C)** | 1.07 (1.00, 1.15) | 1.11 (1.02, 1.21) | 1.04 (0.96, 1.14) | 1.09 (1.01, 1.18) | 1.04 (0.94, 1.16) |
| **P99(11°C)** | 2.73 (2.04, 3.66) | 3.36 (2.32, 4.87) | 2.29 (1.58, 3.33) | 2.73 (1.96, 3.80) | 2.74 (1.74, 4.31) |

CRR, cumulative relative risks; CI, confidence interval

## 2 Supplementary Figures

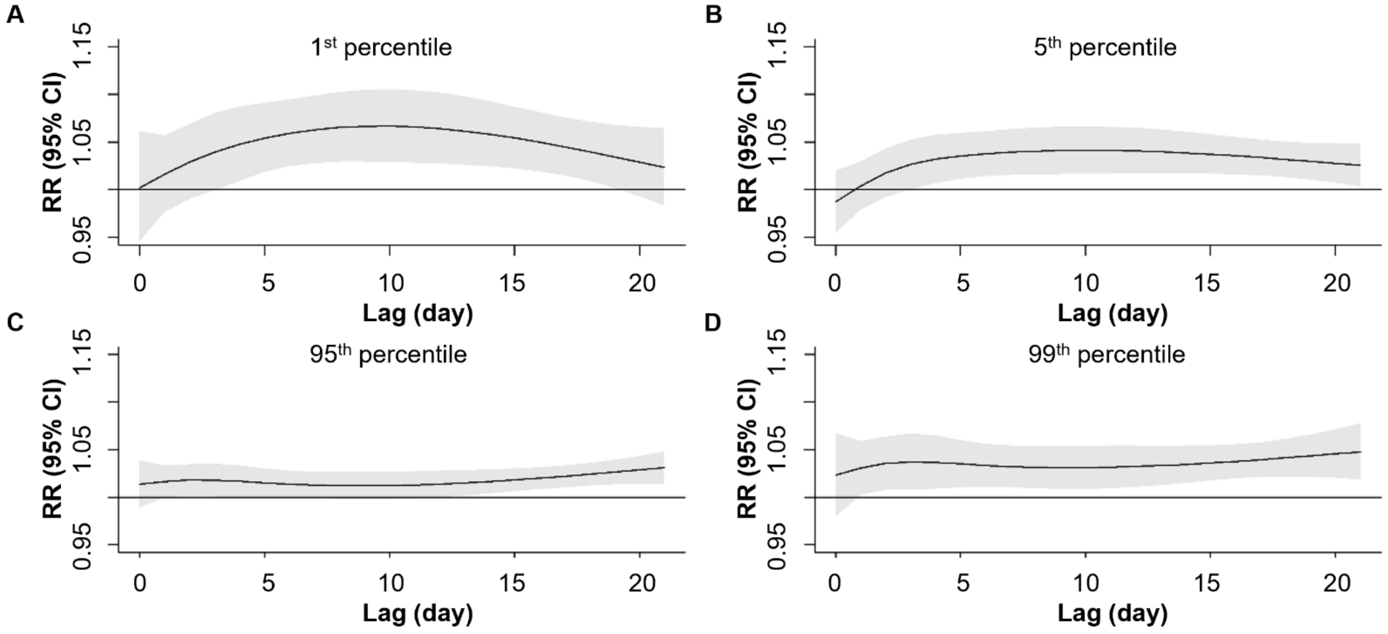


**Supplementary Figure 1**. Lag-response curves for neighboring-day mean temperature difference ($DTDmean_1$) at $1^{st}$, $5^{th}$, $95^{th}$ and $99^{th}$ percentile. Shaded areas represent 95% confidence intervals for relative risks (RR).

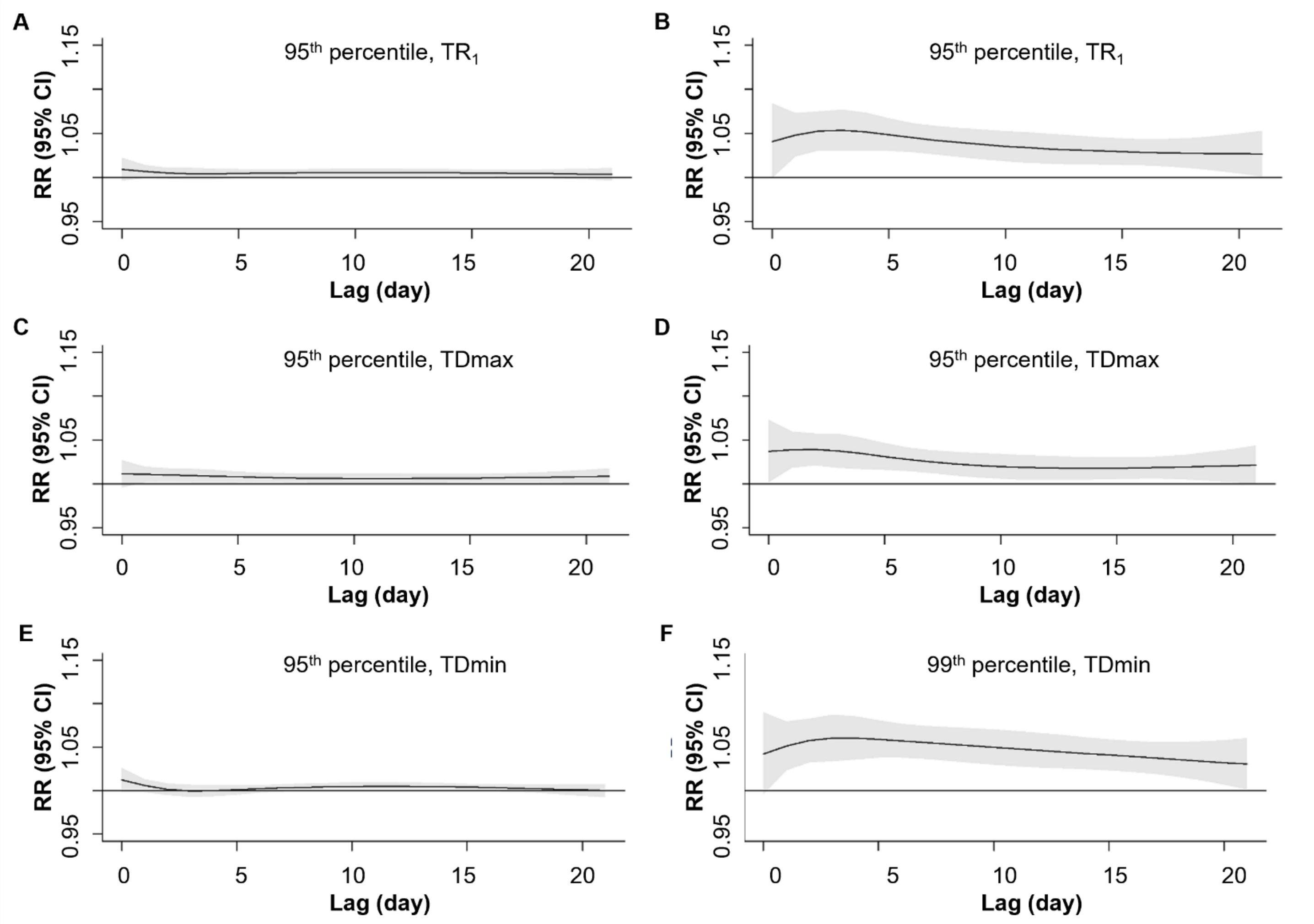


**Supplementary Figure 2**. Lag-response curves for 1-day temperature range ($TR_1$, A and B), maximum-mean temperature difference (TDmax, C and D) and mean-minimum temperature difference (TDmin, E and F) at the 95$^{th}$ and 99$^{th}$ percentile. Shaded areas represent 95% CI for relative risks (RR).

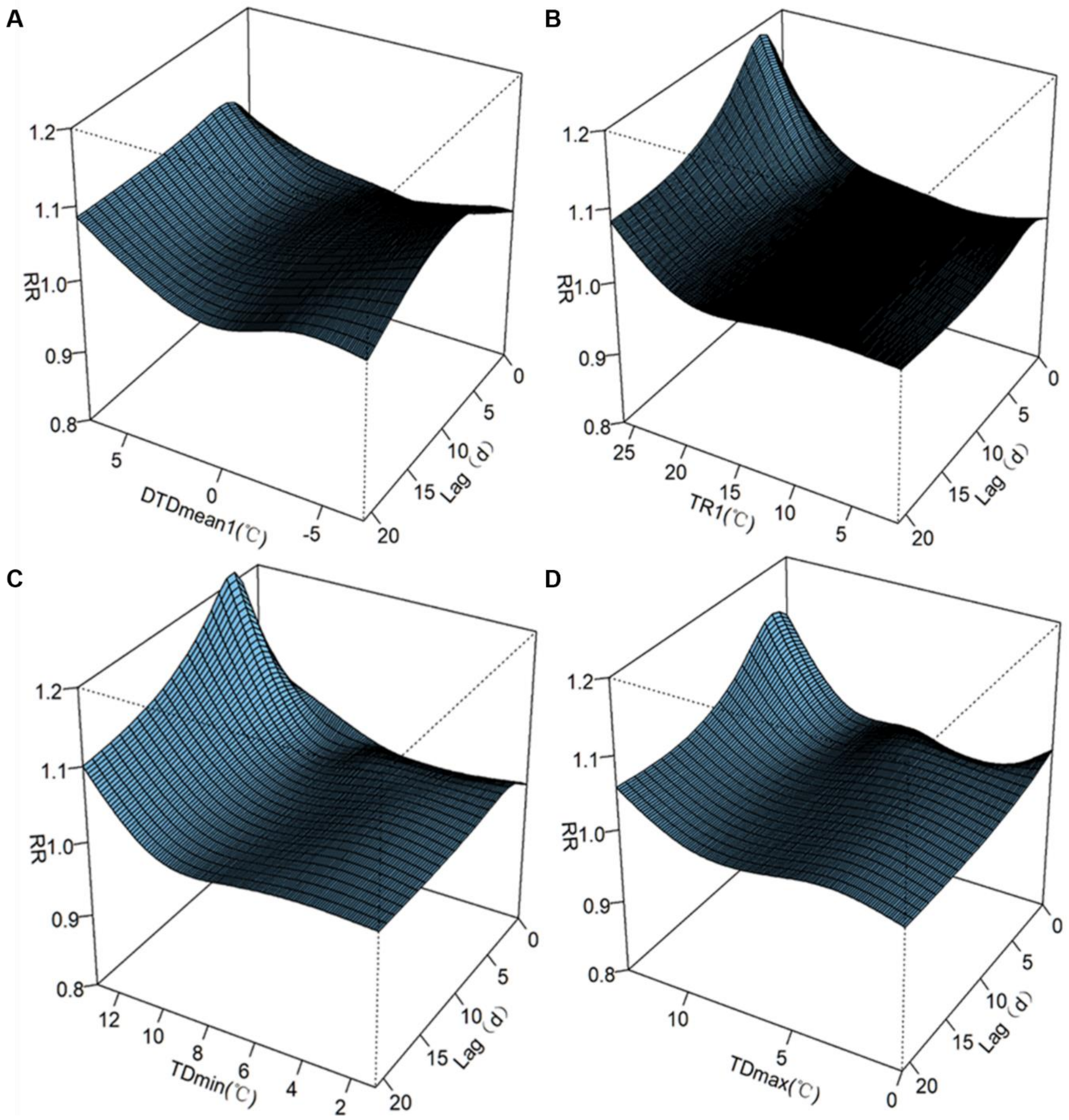


**Supplementary Figure 3**. 3D mapping of the association between relative risk AMI hospitalization and neighboring-day mean temperature difference (DTDmean$_1$, A), 1-day temperature range (TR$_1$, B), Maximum-mean temperature difference (TDmax, C) and Mean-minimum temperature difference (TDmin, D) over the 21-day lag period.

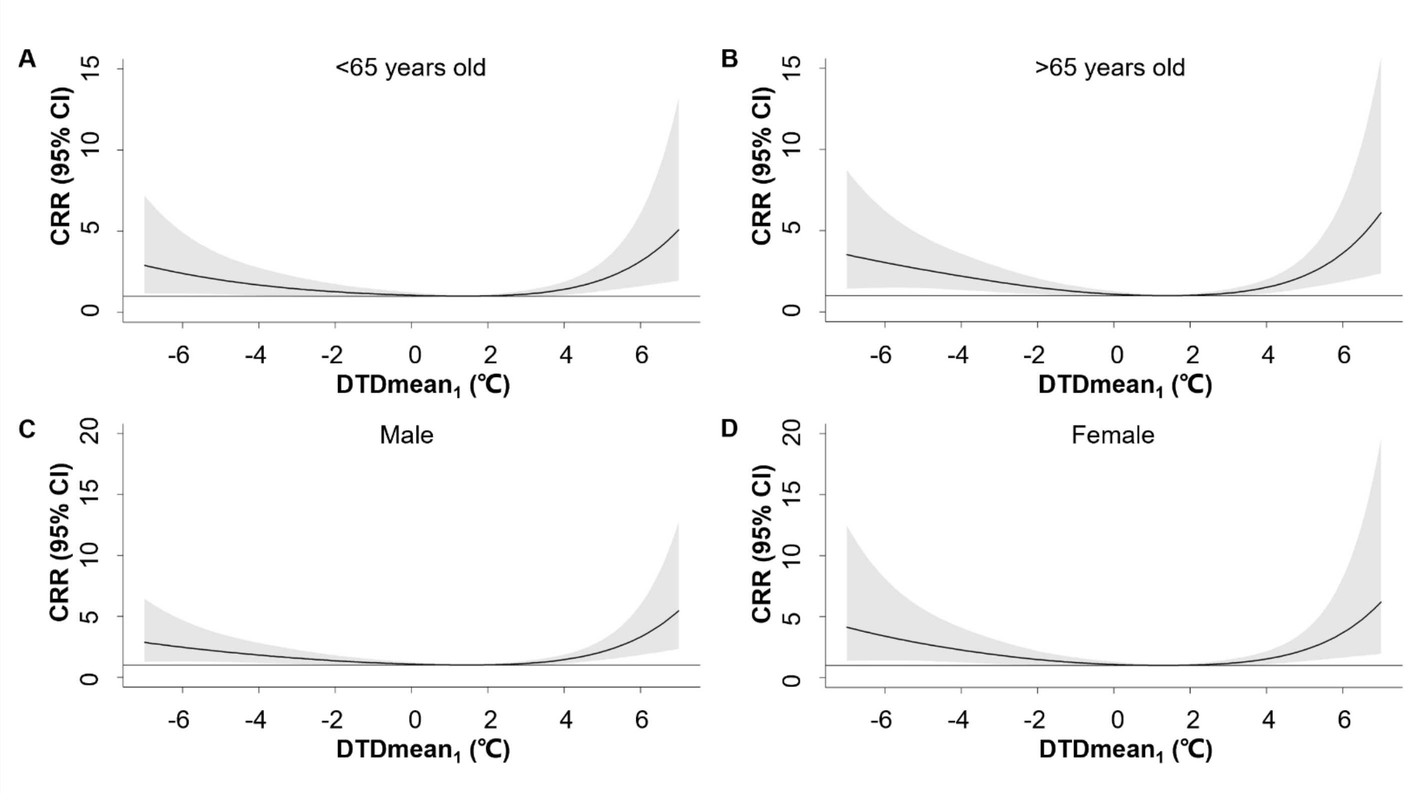


**Supplementary Figure 4**. Exposure-response associations between neighboring-day mean temperature difference (DTDmean$_1$) and cumulative relative risks (CRR) for AMI hospitalization stratified by age (A, B) and sex (C, D). Shaded areas represent 95% CI.

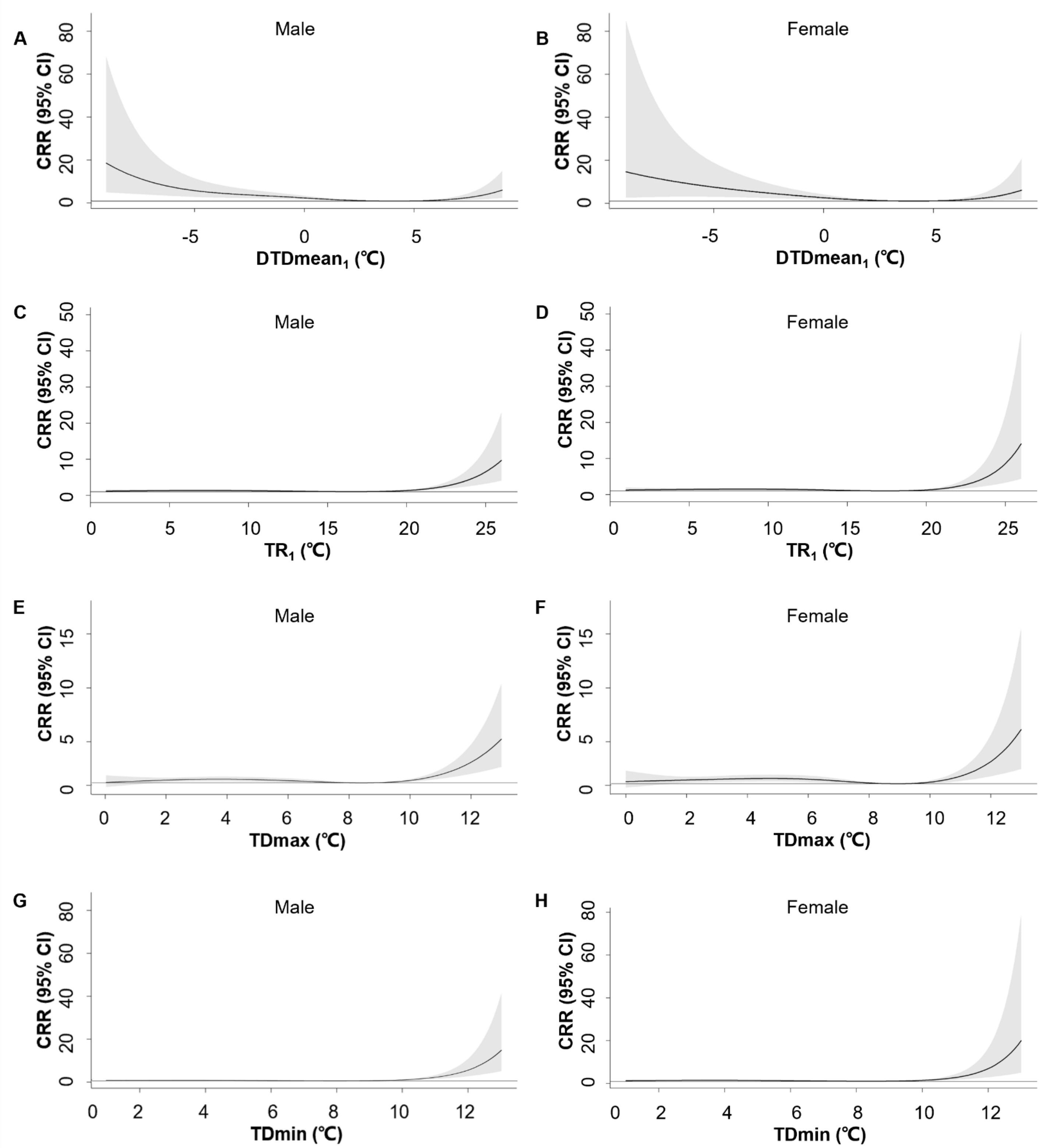


**Supplementary Figure 5.** Exposure-response associations between DTDmean$_1$ (A, B), TR$_1$ (C, D), TDmax (E, F), TDmin (G, H) and CRR (shaded 95% CI) for AMI hospitalization stratified by gender.
